# Supplementary material for: Hidden Partner of Immunity: Microbiome as an Innovative Companion in Immunotherapy
Source: Int J Mol Sci. 2025 Jan 20;26(2):856. doi: 10.3390/ijms26020856 (PMC11765694; doi:10.3390/ijms26020856)
Supplement: Supplementary file 1 [file ijms-26-00856-s001.zip › ijms-3402686-supplementary.pdf]

Supplement Table S1.

ClinicalTrials.gov ID	Official Title	Study type / Phase	Objective	Disease	Related immunotherapy	Status	Location
NCT05725720	Role of the Gut Microbiome in the Outcome of Diffuse Large B-Cell Lymphoma Patients Treated With CAR-T Cell Therapy	Observational	To do characterization of the compositional and functional modifications of gut microbiome in patients affected by lymphoma undergoing therapy with CAR-T cells from baseline until the restaging after 18 months from the CAR-T cell infusion	Diffuse Large B Cell Lymphoma	CAR-T cell	Recruiting	Italy
NCT04691284	Microbiome in Cancer Patients Undergoing High Dose Chemotherapy With Stem Cell Transplantation	Interventional / Not Applicable	To analyze the correlation between microbiome changes after hematopoietic stem cell transplantation/CAR-T therapy and adverse transplant outcomes, such as infectious complications, GvHD, and disease relapse, to explore predictive markers for immune recovery and therapeutic response.	Hematologic neoplasms	CAR T-cell	Recruiting	Slovakia
NCT05621096	Feasibility of Low Dose Radiation as Bridging Therapy for Lisocabtagene Maraleucel in Relapsed B-Cell Non-Hodgkin Lymphoma	Interventional / Phase 1	To assess the feasibility and safety of radiation and CAR-T cell therapy, evaluate response, progression-free survival (PFS), duration of response (DOR), overall survival (OS), and the rate of prolonged cytopenias, and to bank PBMC, serum, and stool samples for future research, while exploring the impact of radiation therapy on local control of disease sites.	B-Cell Non-Hodgkin Lymphoma	CD19 CAR-T cell	Recruiting	United States
NCT06610344	Preliminary Investigation of β-hydroxybutyrate Supplementation for Lymphoma Patients Receiving Anti-CD19 CAR T-cells	Interventional / Not Applicable	To evaluate the feasibility and safety of BHB supplementation in patients receiving anti-CD19 CAR T-cell therapy for lymphoma and to determine if BHB supplementation induces changes in the gut microbiome and peripheral blood mononuclear cells (PBMCs).	Large B-cell Lymphoma	CD19 CAR T-cells	Not yet recruiting	United States
NCT04281797	Intestinal Microbiome Composition Dynamics Over the Course of Kidney Transplantation, Liver Transplantation, Allogeneic Hematopoietic Stem Cells and Mesenchymal Stem Cells Transplantation	Observational	To investigate the dynamic changes in the gut microbiome during kidney, liver, xenogeneic hematopoietic stem cell, and mesenchymal stem cell transplantation.	Solid organ and stem cell transplant	Mesenchymal stem cells	Unknown status	Belarus
NCT02234921	A Pilot Study of DPV-001 DRibble Vaccine With Imiquimod in Advanced Prostate Cancer	Interventional / Phase 1	To evaluate the safety and tolerability of the DRibble vaccine, cyclophosphamide, imiquimod, and Cervarix in castration-resistant prostate cancer, and to investigate the correlation between microbiome characteristics and prostate cancer response	Adenocarcinoma of the Prostate	Cancer vaccine	Completed	United States
NCT03821272	A Phase I/II Clinical Trial of PepCan in Head and Neck Cancer Patients in Remission to Reduce Recurrence Regardless of HPV Status	Interventional / Phase 1, Phase 2	To evaluate the safety and efficacy of administering 7 doses of PepCan or placebo over 24 months in patients with remission of head and neck cancer, assessing safety, efficacy in reducing cancer recurrence, immune response, and changes in the microbiome.	Head and Neck Cancer	Cancer vaccine	Active, not recruiting	United States
NCT03839862	Faecal Analyses in Spondyloarthritis Therapy: A Prospective Observational Study of the Intestinal Microbiome in Patients With Spondyloarthropathy Receiving TNF-inhibition.	Observational	To evaluate if successful treatment of spondylarthropathy coincide with specific changes in the gut flora.	Spondylarthropathy, Ankylosing Spondylitis	Anti-TNF antibody	Recruiting	Sweden
NCT03775824	Faecal Analyses in Rheumatoid Arthritis Therapy: An Prospective Observational Study of the Intestinal Microbiome in Patients With Rheumatoid Arthritis Receiving Immunosuppressive Therapy	Observational	To evaluate the intestinal microbiome and disease activity in patients with rheumatoid arthritis receiving immunosuppressive therapy	Rheumatoid Arthritis	Anti-TNF antibody	Completed	Sweden
NCT05781152	Clinical, Imaging, and Endoscopic Outcomes of Children Newly Diagnosed With Crohn's Disease	Interventional / Phase 4	To understand the factors that determine whether a child with Crohn's disease achieves complete healing of intestinal inflammation after anti-TNF therapy.	Crohn's Disease	Anti-TNF antibody	Recruiting	United States
NCT05542459	Multi-omics to Predict Responses to Biologics in Inflammatory Bowel Disease	Observational	To evaluate the efficacy and safety of biologics in inflammatory bowel disease patients and to identify predictive biomarkers for therapeutic response and safety	Crohn Disease, Ulcerative Colitis	Anti-TNF antibody , anti-IL-12/IL-23	Unknown status	China
NCT04378621	Treatment of Inflammation Versus Hand Training to Prevent and Revert Neuropsychiatric Comorbidity in Patients With Rheumatoid Arthritis	Interventional	To examine how rheumatoid arthritis affects brain structures and whether anti-inflammatory treatments targeting TNF-α or JAK, or hand-focused physical training, positively impact neuropsychiatric symptoms and brain morphology in RA patients.	Rheumatoid Arthritis	TNF-α inhibitors	Active, not recruiting	Sweden
NCT03681652	Post-Operative Crohn's Disease Outcome in Children (The POPCORN Trial): a Prospective Comparative Non-interventional Open Study	Observational	To examine the effect of prophylaxis therapy on clinical and endoscopic disease recurrence in children with Crohn's Disease (CD) following ileo-cecal resection.	Crohn Disease	Anti-TNF Drug	Recruiting	Israel
NCT04859088	Combining Partial Enteral Nutrition With Biologics to Optimise Induction and Maintenance Therapy for Adults With Active Ileocolonic Crohn's Disease	Interventional	To investigate whether replacing half of a habitual diet with specialized milkshakes enhances the effectiveness of biologic treatment in adults with Crohn's disease.	Crohn Disease	TNFα antagonists	Recruiting	United Kingdom
NCT02499263	A Prospective Multicenter Study to Observe the Effectiveness on Ulcerative Colitis and Predictive Factors of Clinical Response in Korean Patients Treated With Adalimumab (EUREKA Study)	Observational	To evaluate the effectiveness and safety of adalimumab treatment in participants with ulcerative colitis over a 56-week period.	ulcerative colitis	Anti-TNF antibody	Completed	Korea, Republic of
NCT03266471	Cytokines and Genes in Therapeutic Response in Crohn's Disease	Observational	To evaluate longitudinal changes in inflammatory cytokines and pharmacogenetic factors associated with treatment response to anti-cytokine agents in individuals with inflammatory bowel disease.	Crohn's disease, ulcerative colitis	anti-TNF antibody	Completed	United States
NCT03209232	Infliximab Accelerated Induction for Moderate to Severe Ulcerative Colitis in Children (INDUCE) Trial	Interventional	To examine the effects of an accelerated infliximab induction schedule compared to standard induction on clinical remission rates in children with moderate to severe ulcerative colitis.	Ulcerative Colitis	anti-TNF antibody	Terminated	Israel
NCT05931458	ADVANCED-UC TRIAL: AppenDectomy Vs ANti TNF-a in Inducing Clinical and EnDoscopic Remission in Left-sided Ulcerative Colitis - A Randomized Clinical Trial	Interventional	To evaluate the impact of appendectomy on the clinical course of ulcerative colitis in patients with left-sided colitis who are candidates for anti-TNF therapy	ulcerative colitis	anti-TNF antibody	Not yet recruiting	-
NCT03819296	Role of Microbiome in the Realm of Immune-Checkpoint Inhibitor Induced GI Complications In Cancer Population	Interventional / Phase 1, Phase 2	To study the role of the gut microbiome and the effectiveness of fecal transplants in managing medication-induced gastrointestinal complications, such as colitis, in patients with melanoma or genitourinary cancer receiving immune-checkpoint inhibitor therapy.	medication-induced gastrointestinal	ICI, Immunosuppressive agent	Recruiting	United States
NCT03358693	Systematic Profiling of Anti-cytokine Signatures in the Treatment of Chronic Inflammatory Skin Disorders	Observational	To evaluate a systems medicine approach to identify regulatory networks and their disruptions in psoriasis and atopic dermatitis	Atopic Dermatitis, Psoriasis	anti-TNF antibody, anti-IL-4, IL-13 antibody / anti-IL-12, IL-23 antibody	Recruiting	Germany
NCT04505410	A Randomized Control Study to Examine the Influence of a Healthy Diet on Moderate to Severe Ulcerative Colitis Patients Undergoing Second Line Induction With Biological Medications Tofacitinib, Ustekinumab or Vedolizumab	Interventional / Phase 3	To determine whether a diet intervention (the Fasting Mimicking diet) will help induce clinical and biochemical response to tofacitinib therapy or second line biologic therapy with ustekinumab or infliximab in patients with ulcerative colitis.	Ulcerative Colitis	anti-TNF antibody, anti-IL-12, IL-23 antibody	Completed	United States
NCT04107168	An Observational Study to Evaluate the Microbiome as a Biomarker of Efficacy and Toxicity in Cancer Patients Receiving Immune Checkpoint Inhibitor Therapy	Observational	To investigate how the microbiome correlates with efficacy and toxicity of immune checkpoint inhibitors in patients with advanced cancer.	Melanoma, Renal Cancer, Lung Cancer	anti- VEGF antibody	Recruiting	United Kingdom
NCT03818061	A European, Multi-centre Phase II Trial of Atezolizumab and Bevacizumab in Patients With Recurrent or Metastatic Squamous-cell Carcinoma of the Head and Neck (HNSCC)	Interventional / Phase 2	To assess the clinical and biological effects of Atezolizumab combined with Bevacizumab in advanced previously treated squamous-cell carcinoma of the head and neck (HNSCC).	Head and Neck Neoplasms	anti- VEGF antibody	Active, not recruiting	France
NCT04054908	Gut Microbiome and Oral Fluoropyrimidine Study in Patients With Colorectal Cancer	Observational	To investigate the alterations in the gut microbiome that occur during the course of treatment for colorectal cancer	colorectal cancer	anti- VEGF antibody	Completed	University of California, San Francisco
NCT03694262	An Open Label, Non-Randomized Multisite Phase II Trial Combining Bevacizumab, Atezolizumab and Rucaparib for the Treatment of Previously Treated Recurrent and Progressive Endometrial Carcinoma	Interventional / Phase 2	To demonstrate the efficacy and safety of the combination of rucaparib, bevacizumab and atezolizumab in recurrent, progressive endometrial carcinoma.	Endometrial Cancer, Uterine Carcinosarcoma	anti- VEGF antibody	Completed	United States
NCT06551272	Microbiota Modification for Immuno-oncology in Hepatocellular Carcinoma	Interventional / Phase 2	To test the concept of microbiota modification in patients treated with atezolizumab-bevacizumab for advanced HCC	Hepatocellular Carcinoma	Anti-TcdB antibody	Not yet recruiting	France
NCT05312398	CAPRI 2 GOIM Study: Investigate the Efficacy and Safety of a Bio- Marker-driven Cetuximab-based Treatment Regimen Over 3 Treatment Lines in mCRC Patients With RAS/BRAF wt Tumors at Start of First Line	Interventional / Phase 2	To evaluate the activity and efficacy of cetuximab continuation of treatment for three lines of therapy with rotation of chemotherapy (FOLFIRI, FOLFOX, irinotecan) in mCRC patients, whose tumors remain RAS/BRAF WT	Metastatic Colorectal Adenocarcinoma	anti-EGFR antibody	Active, not recruiting	Italy
NCT05036733	Effects of IL-4R-alpha Inhibition (Dupixent) Inhibition On The Respiratory Microbiome And Immunologic Correlates In Patients With Severe Asthma	Interventional/ Phase 4	To understand biological responses related to dupilumab treatment among severe asthma patients.	Asthma	anti-IL-4, IL-13 antibody	Completed	United States
NCT03293030	Immunogenetic Profiling of Dupilumab for the Treatment of Atopic Dermatitis	Interventional / Phase 4	To examine the effect of dupilumab on the immunologic and genetic environment within atopic dermatitis skin lesions.	Atopic Dermatitis	anti-IL-4, IL-13 antibody	Completed	United States
NCT05099315	Identification of Skin-associated Microbiota in Atopic Dermatitis Patients Undergoing Systemic Therapy	Observational [Patient Registry]	To investigate whether and how the skin microbiome changes in patients with moderate to severe atopic dermatitis during systemic therapy	Atopic Dermatitis	anti-IL-4, IL-13 antibody	Recruiting	Germany
NCT05436535	Longitudinal Endotyping Of Atopic Dermatitis Through Transcriptomic Skin Analysis (ADRN-12)	Interventional / Phase 4	To determine if the type 2-high non-lesional skin (skin tape) endotype is associated with current mild versus moderate-severe AD disease.	Atopic Dermatitis	anti-IL-4, IL-13 antibody	Recruiting	United States
NCT03389893	Effect of Dupilumab (Anti-IL4Rα) on the Host-Microbe Interface in Atopic Dermatitis, Dupilumab Study	Interventional / Phase 4	To understand the effect that T helper 2 (Th2) blockade has on well-described pathophysiological features of Atopic Dermatitis	Atopic Dermatitis	anti-IL-4, IL-13 antibody	Terminated	United States
NCT05061329	The Importance of the Nasal Microbiome in Transmission and Disease	Observational [Patient Registry]	To explore the relationship between the microbiome, immunology, and olfactory dysfunction.	Chronic Rhinosinusitis (Diagnosis), Anosmia, COVID-19	anti-IL-4, IL-13 antibody	Recruiting	Denmark
NCT04779762	Stelara and CDED Diet Trial for Crohn''s Disease	Interventional	To evaluate the added benefit of treatment with Ustekinumab combined with CDED in anti TNF exposed patients compared to treatment with Ustekinumab alone in term of achieving remission.	Crohn Disease	anti-IL-12, IL-23 antibody	Not yet recruiting	Israel
